# Impact of Enzymatic Degradation of the Endothelial Glycocalyx on Vascular Permeability in an Awake Hamster Model

**DOI:** 10.1155/2012/842545

**Published:** 2012-06-26

**Authors:** S. A. Landsverk, A. G. Tsai, P. Cabrales, M. Intaglietta

**Affiliations:** ^1^Department of Bioengineering, University of California, San Diego, La Jolla, CA 92093, USA; ^2^Department of Anesthesiology, Oslo University Hospital, 0424 Oslo, Norway

## Abstract

*Background*. The inside of the endothelium is covered by a glycocalyx layer, and enzymatic degradation of this layer induces vascular leakage *ex vivo*. We hypothesized that enzymatic degrading of the glycocalyx in an *in vivo*, whole body model, would induce plasma leakage and affect the microcirculation. *Methods*. Golden Syrian hamsters were divided into an enzyme (hyaluronidase) and a control group. Mean arterial pressure (MAP), heart rate (HR), hematocrit (Hct), base excess (BE), and plasma volume were obtained before, 45 and 120 min after enzyme/saline treatment. Plasma volume was evaluated by the distribution volume of indocyanine green and the microcirculation by functional capillary density (FCD). The enzymatic effect was determined by measuring plasma levels of hyaluronan (HA). *Results*. There were no differences in MAP, HR, Hct, and BE between the two groups. Enzyme treatment did not induce changes in plasma volume but reduced FCD. There was a 50–100-fold increase in plasma HA, but no relationship was found between HA levels and plasma volume or FCD. *Conclusion*. Vascular leakage was not confirmed in an *in vivo*, whole body model after degradation of the endothelial glycocalyx. The microcirculation was affected, but no relationship between plasma levels of HA and FCD was seen.

## 1. Introduction

Increased vascular permeability is often seen in surgical and critical care patients [1] and has, among other mechanisms, been attributed to damage of the endothelial glycocalyx. The importance of an intact glycocalyx layer to prevent vascular leakage has been demonstrated by experimental degradation of this layer in isolated hearts or blood vessels, and *in vivo* using genetically modified mice predisposed to atherosclerosis [2–5]. In these experimental models, different approaches to preserve the glycocalyx has also been evaluated [6, 7]. Hyperglycemia, ischemia, and inflammation are associated with degradation of the endothelial glycocalyx [8, 9]. Based on this knowledge, clinical recommendations have been given for the preservation of the glycocalyx during surgery, to avoid pathological fluid and protein shifts [10]. As far as we are aware of, increased vascular permeability with decreased plasma volume or tissue edema after enzymatic degradation of the glycocalyx has not been demonstrated in an *in vivo*, whole body (wild-type) model. A previous study performed in our laboratory found no reduction in plasma volume after enzymatic degradation of the glycocalyx in hamsters, whereas the microcirculation was affected, demonstrated by a reduction of functional capillary density (FCD) [11]. The effect of hyaluronidase on the endothelial glycocalyx was not evaluated in that study and the plasma volume tracer used, Dextran 40 kDA, has been criticized [12]. 

The aim of the present study was to evaluate plasma leakage and impairment of the microcirculation by enzymatic degrading the endothelial glycocalyx in an awake hamster model. A plasma volume tracer, indocyanine green (ICG), suitable for repetitive measurements was used, and as vascular leakage is time dependent, the observation period was extended. Changes in plasma volume and FCD could then be related to enzymatic effects measured by the total amount of hyaluronan (HA) released into the circulation. 

## 2. Materials and Methods

### 2.1. Animals

Male Golden Syrian hamsters, 6-7 weeks old, weight 58–66 gr (Charles River Laboratories, Boston, MA), were used in a hamster window chamber model. The protocol was approved by the local animal subjects committee and was in accordance with the Guide for the Care and Use of Laboratory Animals (National Research Council, 1996). The hamster window chamber model allows the study of the skin microcirculation, to infuse drugs and to collect blood samples without the influence of anesthesia [13]. The implantation of the chamber window and catheters in the carotid artery and jugular vein (PE50/PE10) were performed in two separate procedures during anesthesia (Nembutal, 50 mg/kg, intraperitoneal injection, Abbott, Abbott Park, IL). The complete surgical technique is previously described [13]. Experiments were performed 48 hours after the implantation of the catheters, without any influence of anesthetic drugs.

### 2.2. Hemodynamic Parameters and Hematology

Continuous blood pressure was obtained from a carotid artery catheter during the experimental period, giving mean arterial pressure (MAP) and heart rate (HR) (Biopac, Santa Barbara, CA; Spectramed Pressure Transducer). Hemoglobin level was determined spectrophotometrically (B-Hemoglobin, Hemocue, Stockholm, Sweden) and hematocrit was measured from centrifuged arterial blood samples using heparinized capillary tubes. Analysis of PaO_2_, PaCO_2_, base excess and pH was determined from arterial blood, collected in heparinized capillary tubes (Blood Chemistry Analyzer 248, Bayer, Norwood, MA). 

### 2.3. Plasma Levels of Albumin

Vascular permeability has previously also been measured using transcapillary escape rate of radioactive labeled albumin [14]. An increased leakage of albumin due to degradation of the endothelial glycocalyx could reduce the total amount of plasma albumin. Plasma levels of albumin were determined (Vetscan VS2, Abaxis Inc. Union City, CA) before and 45 min after enzyme or saline was given, in a separate group of hamsters (*n* = 6).

### 2.4. Distribution Volume of Indocyanine Green

ICG has been used as a method to measure plasma volume [15]. The advantage of ICG relates to its low toxicity, rapid distribution, and clearance allowing for repetitive measurements [16]. Whole blood was used to determine dye absorption. A calibration curve was made from two known concentrations of dye in blood from one hamster. The circulation time of ICG was based on multiple samples from pilots. Baseline absorption was obtained by taking 10 *μ*L samples from the arterial line before each measurement. 0.1 mg of ICG, diluted in 0.1 mL sterile water, was then given intravenously. 10 *μ*L blood was then collected from the arterial line at 3, 4, 5, and 6 min after the injection of the dye and placed in a cuvette with 100 *μ*L deionized water before each measurement. To reduce dye contamination and minimize blood loss, the length of the arterial line was adjusted so that taking 2 drops of blood before each sample would ensure that the sample taken was from the circulation, and not from the catheter. In addition, the tip of the catheter was cleaned between each sample. The cuvette was analyzed in a spectrometer (Lambda 20, Perkin Elmer, Waltham, MA), absorption measured at 800 and 880 nm. Mixing of the cuvette, timing from obtaining samples to analysis was performed similarly each time. A monoexponential extrapolation was performed to calculate absorption at time zero. Measurements were included only if *r* ^2^ ≥ 0.9.

### 2.5. Functional Capillary Density (FCD)

FCD was evaluated microscopically as the number of capillary vessel with erythrocytes passing in the visual field during one minute. 10 visual fields were counted and the average value calculated. Each visual field was identified in a way that repeating measurements could be obtained at the same location. 

### 2.6. Plasma Levels of Hyaluronan

Plasma levels of hyaluronan were determined by hyaluronan—Enzyme Linked Immunsorbent Assay kit (HA-ELISA) (Echelon Bioscience Inc., Salt Lake City, UT). Arterial blood was collected at baseline, 45, 60, and 120 min after hyaluronidase was given, using a heparinized capillary tube. After centrifuging, 10 *μ*L of plasma was obtained with a micropipette and then transferred to an Eppendorf tube and stored at −80°C, until analysis.

### 2.7. Experimental Setup and Protocol

**Figure 1:**
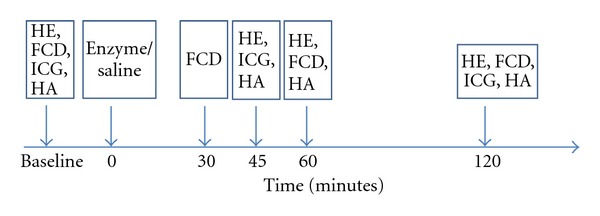
The time line shows the experimental setup. Baseline registrations includes hemodynamic and hematologic data (HE), functional capillary density (FCD) the distribution volume of indocyanine green (ICG) and plasma hyaluronan (HA). Enzyme or saline was given at time zero. Following measurements are indicated with an arrow.

14 animals were divided in two groups, receiving either Streptomyces hyaluronidase (Sigma-Aldrich, St. Louise, MO) or saline. In a separate group of animal (*n* = 6), hyaluronidase or saline was given to determine the impact on albumin levels. On the day of the experiment, the hamster was placed in a restraining tube with a longitudinal slit for the chamber window. The anesthetized animal was made to adapt to the new environment for 30 min. Baseline values of MAP, HR, hematology, including levels of HA in plasma and the distribution volume of ICG were then obtained. A bolus of 100 units of hyaluronidase (0.1 mL) or a similar volume of saline was infused after baseline measurements. This represents time zero, in the time line shown in Figure 1. The distribution volume of ICG was measured at 45 and 120 min. FCD was obtained at 30 and 60 min and before the distribution volume of ICG at 120 min. Hemodynamic parameters and hematology were obtained at 45, 60, and 120 min, together with blood samples for HA measurement. 

### 2.8. Data Analysis and Statistics

**Figure 2:**
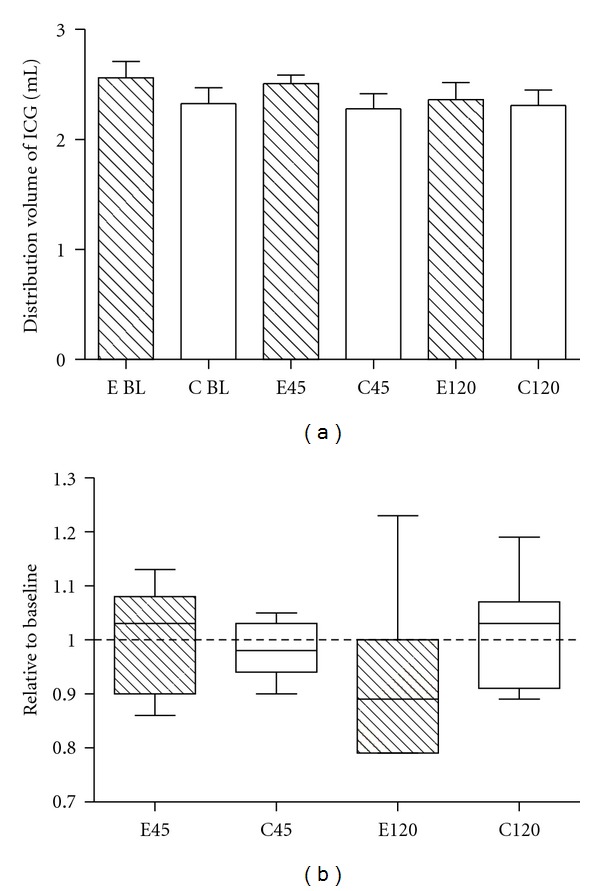
Effect of enzymatic degradation of the glycocalyx on the distribution volume of indocyanine green (ICG). (a): There was no difference within or between the enzyme group (E-shaded bars) and the control group (C-open bars), at baseline (BL) and after 45 or 120 minutes. (b): There is no difference in values relative to baseline for the distribution volume of ICG after 45 minutes: Enzyme group (E45) and control group (C45), and after 120 minutes, enzyme group (E120) and control group (C120).

**Figure 3:**
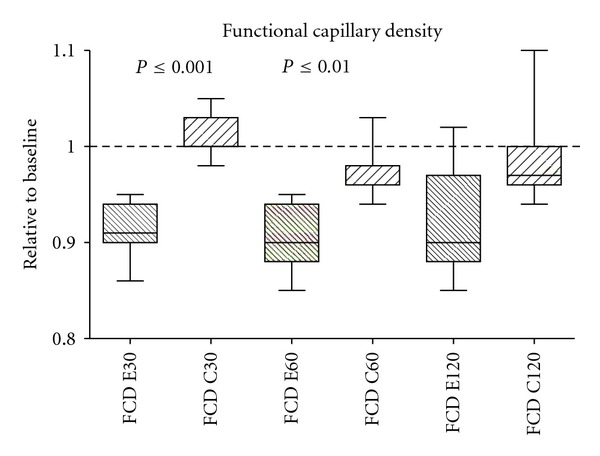
Effect of enzymatic degradation of the glycocalyx on functional capillary density (FCD). Data shown as values relative to baseline. Significant changes were found, between the enzyme group (E30) and control group (C30) after 60 minutes (E60, C60). After 120 minutes (E120, C120) there was a non significant trend (*P* = 0.07).

Values are given as mean and standard deviation, unless, otherwise, stated. Data on distribution volume of ICG- and FCD are also given as relative values to baseline. A value of 1.0 then refers to zero change from baseline. In Figures 2(b) and 3, data are shown as box plots. The horizontal line within the box represents median value. The upper and lower limit of the box represents the 75th and 25th% percentile and the upper and lower whisker represents the 95th and 5th%. Comparisons within groups were performed with a one-way ANOVA, with post hoc analyses performed with the Bonferroni's multiple-comparison tests. Comparisons between the groups at each time point regarded were performed with an unpaired *t*-test. All statistics were calculated using GraphPad Prism 4.01 (GraphPad Software, San Diego, CA). Changes were considered statistically significant if *P* < 0.05.

## 3. Results

A total of 20 animals were used in this study. An enzyme group (*n* = 7, weight 63.4 ± 2.3 g) received Streptomyses hyaluronidase and a control group (*n* = 7, weight 61.4 ± 3.6 g) was given the same amount of saline. There was an estimated blood loss of 0.35 mL, and an infusion of 0.6 mL saline between each measurement of the distribution volume of ICG. The impact of enzymatic degradation of the glycocalyx on plasma albumin levels was tested in a separate group of animals (*n* = 6).

### 3.1. Hemodynamic Parameters and Hematology

There was no significant difference between the groups at the three different time points. Within the groups, there were no differences in pH, BE, PO_2_, and PCO_2_. There was trend to lower MAP from baseline to 120 min in the enzyme group and a similar, but significant reduction in the control group. HR was decreased between 60 and 120 min in the control group. Hct was reduced significantly from baseline values to 120 min in both groups. Data are shown in Table 1.

### 3.2. Levels of Albumin after Hyaluronidase/Saline

In the separate group of hamsters (*n* = 6), there was no difference in levels of plasma albumin before and at 45 min in the group with enzyme treatment (4.5 ± 0.3 gm/dL versus 4.5 ± 0.2 gm/dL) or in animals receiving saline.

### 3.3. Distribution Volume of Indocyanine Green

No differences at baseline values were found between groups. 45 min after enzyme or saline treatment, no changes in distribution volume were found (relative values to baseline 0.99 ± 01 versus 0.98 ± 0.05, *P* = 0.8). After 120 min, there was a trend in reduction of ICG distribution volume in the enzyme group, but there was no significant difference between the two groups (relative values to baseline 0.93 ± 0.16 versus 1.01 ± 0.11, *P* = 0.25). Data are shown as absolute values and relative values to baseline in Figure 2.

### 3.4. Functional Capillary Density

FCD was significant lower in the enzyme group than in the control group at 30 and 45 minutes. There was nonsignificant trend (*P* = 0.07) at 120 minutes. Data are shown as values relative to baseline in Figure 3.

### 3.5. Plasma Levels of HA

**Figure 4:**
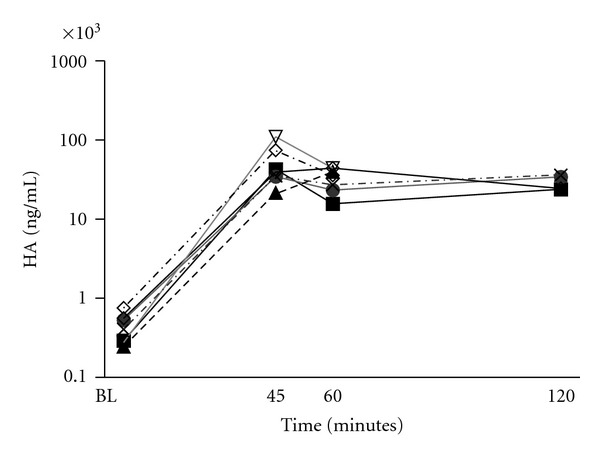
Plasma levels of hyaluronan (HA) for the 7 animals treated with hyaluronase displayed on a logarithmic axis. Plasma level of HA from each animal is indicated by a single line based on measurements obtained at baseline (BL), after 45, 60, and 120 minutes. At 120 minutes, HA was measured in 4 animals.

There was a 50–100 folds increase of HA levels after hyaluronidase treatment. Plasma levels of HA are shown in Figure 4.

### 3.6. Relation between Levels of Hyaluronan and Distribution Volume ICG

**Figure 5:**
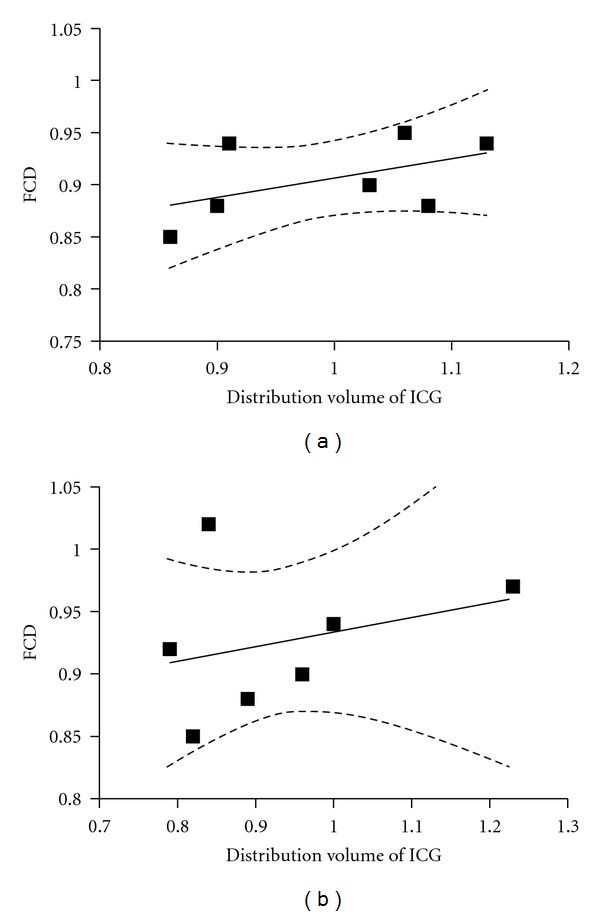
(a): Correlation between functional capillary density (FCD) and the distribution volume of indocyanine green (ICG) at 45 minutes (a) and 120 minutes (b). FCD obtained at 30 minutes was used for the correlation at 45 minutes. The confidence intervals for the regression line (the two dotted lines) indicates the lack of correlation at any of the time points.

There was no significant relationship between plasma volume and FCD at 45 and 120 min (Figures 5(a) and 5(b)) as the confidence intervals for the regression lines were not significantly different from zero. 

### 3.7. Relation between Plasma Levels of HA and Distribution Volume of ICG and FCD

**Figure 6:**
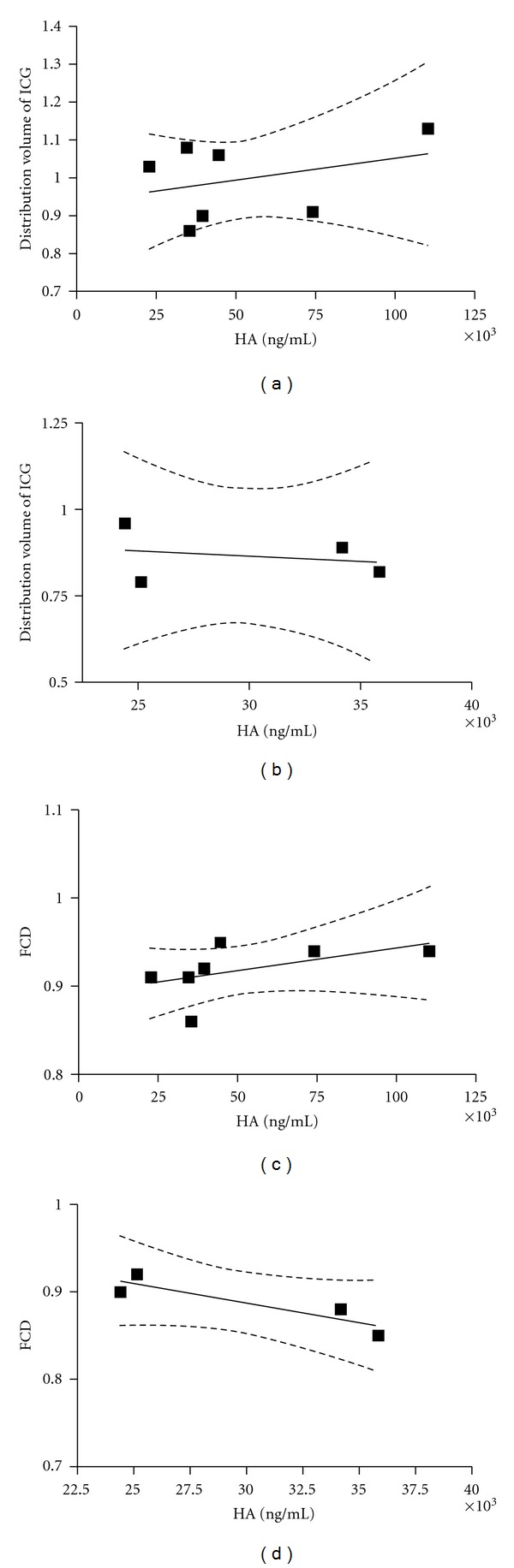
Correlation between plasma hyaluronan (HA) and the distribution volume of indocyanine green (ICG) after 45 and 120 minutes ((a) and (b)). The correlation between functional capillary density (FCD) is shown after 45 and 120 minutes ((c) and (d)). The confidence interval for the regression lines are marked as dotted lines and indicate no correlation.

There was no significant relationship between levels of HA and the distribution volume of ICG at 45 and 120 min (Figures 6(a) and 6(b)) or with FCD (Figures 6(c) and 6(d)). The confidence intervals for all the regression lines were not significantly different from zero. 

## 4. Discussion

The main finding in this study was that enzymatic degradation of the endothelial glycocalyx in an *in vivo*, whole-body model, did not induce leakage of plasma or albumin. The microcirculation was affected, demonstrated by a significant reduction of FCD. However, there was no clear relationship between the amount HA released into the circulation and the distribution volume of ICG or FCD. 

The clinical consequences of increased vascular permeability are tissue edema and hypovolemia, associated with reduced tissue oxygenation and impairment of organ function. Thus, increased vascular permeability represents a significant clinical challenge. Increased attention has been given to the endothelial glycocalyx and its role in regulating vascular permeability [17]. Previous studies have been performed in a variety of experimental settings, from cultured cells, *ex vivo* isolated organs or vessels, genetically modified animals, mainly focusing on the permeability of proteins and tracers within the glycocalyx layer. Conflicting results on glycocalyx thickness from *in vitro* and *ex vivo* studies also indicate that the integrity of this structure is dependent on experimental conditions [18]. Surprisingly, few studies have addressed the clinical consequences of increased vascular permeability, such as plasma leakage and tissue edema. Despite this, experimental studies have been used as evidence for clinical recommendations in fluid therapy [10, 19]. Interestingly, the authors of these two reviews draw different conclusions based on the same experimental literature regarding the use of colloids.

The fact that enzymatic degradation had no impact on the distribution volume of ICG was supported by laboratory and hemodynamic data. There was no difference in blood pressure, heart rate, Hct or BE between the enzyme group and the control group indicating an increased vascular leakage after hyaluronidase treatment. 

Our findings are in accordance with a previous study in our laboratory, using the same animal model, but with a different plasma volume tracer, Dextran 40 kDA [11]. These two studies are in contrast to the vascular leakage leading to myocardial edema seen *ex vivo* [4] or reduced plasma volume and proteinuria after 4 weeks of hyaluronidase treatment in Apolipoprotein E-deficient mice [5]. The intensity of the hyaluronidase treatment of the isolated heart or the duration of treatment of the genetically modified vascular wall could explain these conflicting findings. 

We found that FCD was reduced after enzymatic treatment. FCD is one of the most important indicator of tissues perfusion and has been shown to predict survival after shock both in clinical and animal studies [20–22]. Reduction of the circulating plasma volume is one of many factors known to affect FCD. However, in our study, there was no relation between FCD and the distribution volumes of ICG (Figure 5). Previously, several mechanisms for the relationship between FCD and the degradation of the endothelial glycocalyx have been proposed. Impairment of mechanotransduction associated with vasoconstriction, mainly due to loss of hyaluronan from the endothelial glycocalyx [23] was suggested as the most likely mechanism in a study by Zuurbier et al. [24]. However, when microcirculatory vessels diameter and flow was measured before and after hyaluronidase treatment, this was not confirmed [11]. In a study by Luquita et al. [25], increased erythrocyte rigidity was seen with elevated levels of plasma HA. Although there was no clear relationship between FCD and HA levels in the present study, the levels of HA was 50–100 times higher in all animals. Elevated levels of HA are also related to increased viscosity [11]. Thus, increased levels of HA might contribute to reduced FCD seen in our study. 

Detection of components from the glycocalyx, such as syndecan, heparan sulfate, and HA, has previously been used as evidence for enzymatic degradation [9, 26]. By measuring HA in the animals together with the distribution volume of ICG and FCD, we evaluated the relationship between these parameters. The distribution volumes of ICG were similar after 45 min in both group, but with a larger variation seen in the enzyme group compared to the saline group. The standard deviation was almost twice. This pattern was also seen in FCD, although not as clear. A large variation in FCD, arteriolar and venular diameter, and velocity, was also seen in a previous study [11]. As seen in Figure 6, there was no relationship between either the distribution volume of ICG, or FCD and levels of HA. Thus, variability found in both studies cannot be explained by a different response of hyaluronidase to produce HA. 

There are several other factors that can influence our observations. The endothelial glycocalyx is influenced by a wide range of stimuli. The two-step surgical procedure, the implantation of the window chamber one day, and then the catheters two days before the experiment could induce a postoperative response impairing the whole endothelial glycocalyx in both groups, thus reducing the difference after enzyme treatment.

Many baseline values of HA in our study were high, but reasonable based on the hamsters young age and the reduction of food intake postoperatively. Hamster usually drops 3–5 grams after surgery [27]. The surgery and postoperative alterations could also contribute. The increase in plasma levels of hyaluronan after enzymatic treatment was substantial. Even though others have found large increase of glycocalyx components after shedding [9], it is possible that the increase in circulating HA could originate from other sources than the endothelial glycocalyx [28]. Most of HA are found in the extracellular matrix, and the size of the hyaluronidase molecule would allow penetrating the capillary wall. Thus, it is likely that a considerable amount of HA found could be a product of the extracellular matrix. The high plasma levels of HA 2 hours after the enzyme bolus probably reflect that there is no reincorporation of HA into the glycocalyx, and that the plasma levels exceed the capacity of the liver to metabolize HA [29, 30].

Hyaluronidase only degrades parts of the glycocalyx, and the remaining structure could be capable of preventing plasma leakage. As the endothelial glycocalyx regenerates slowly [31], and plasma and protein leakage are time dependent, the time frame of the present study, although longer compared to the previous study [11], could have been too short. Thus, extending the time frame, combining several enzymes [32], including additional experimental methods such as detecting leakage of ICG into to perivascular space using intravital fluorescence microscopy, could be an approach to demonstrate vascular leakage in an *in vivo*, whole-body model in the future. 

## 5. Conclusion

Enzymatic degradation of the endothelial glycocalyx with hyaluronidase does not induce plasma leakage in awake hamsters in a two hours' time frame, but reduces FCD. No relationship between changes in plasma volume or FCD to the amount of HA released into the circulation after enzyme treatment was found.

## Figures and Tables

**Table 1:** Hemodymanical parameters and hematology.

	Baseline		60 minutes		120 minutes	
	Enzyme	Control	Enzyme	Control	Enzyme	Control
MAP	114 (5)	111 (6)	109 (5)	104 (9)	106 (9)	99 (5)^∗^
HR	459 (16)	468 (17)	455 (22)	473 (18)	454 (16)	448 (16)^∗^
Hct	49 (2)	50 (2)	47 (2)	47 (2)	45 (2)^∗∗^	45 (2)^∗∗∗^
Ph	7.37 (0.03)	7.35 (0.04)	7.35 (0.04)	7.32 (0.04)	7.35 (0.02)	7.32 (0.03)
P_a_O_2_	56.7 (9.9)	54.4 (5.7)	61.2 (5.9)	59.5 (8.9)	57.1 (10.9)	59.2 (7.4)
P_a_CO_2_	56.6 (7.5)	59.7 (3.89)	56.1 (7.6)	61.3 (6.6)	55.7 (5.2)	59.2 (2.7)
BE	6.8 (2.8)	6.9 (2.1)	4.4 (1.6)	4.7 (2.3)	4.4 (1.6)	3.9 (2.0)

Values are given as mean and standard deviation. MAP: mean arterial pressure, HR: heart rate, Hct: hematocrit, BE: base excess. Significant differences were seen within the groups from baseline to 120 minutes (MAP and Hct) and from 60 to 120 minutes in the control group (HR). ^∗^ *P* < 0.05, ^∗∗^ *P* < 0.01, ^∗∗∗^ *P* < 0.001.
